# Conjunctival Neutrophils Predict Progressive Scarring in Ocular Mucous Membrane Pemphigoid

**DOI:** 10.1167/iovs.16-19247

**Published:** 2016-10

**Authors:** Geraint P. Williams, Peter Nightingale, Sue Southworth, Alastair K. O. Denniston, Paul J. Tomlins, Stephen Turner, John Hamburger, Simon J. Bowman, S. John Curnow, Saaeha Rauz

**Affiliations:** 1 Academic Unit of Ophthalmology, Centre for Translational Inflammation Research, Institute of Inflammation and Ageing, College of Medical and Dental Sciences, University of Birmingham, Birmingham, United Kingdom; 2 Birmingham and Midland Eye Centre, Sandwell and West Birmingham Hospitals NHS Trust, Birmingham, United Kingdom; 3 Wellcome Trust Clinical Research Facility, University Hospitals Birmingham NHS Foundation Trust, Birmingham, United Kingdom; 4 School of Dentistry, College of Medical and Dental Sciences, University of Birmingham, Birmingham, United Kingdom; 5 Department of Rheumatology, University Hospitals Birmingham NHS Foundation Trust, Birmingham, United Kingdom

**Keywords:** impression cytology, pemphigoid, progression, autoimmune disease, fibrosis and neutrophils

## Abstract

**Purpose:**

Ocular mucous membrane pemphigoid (OcMMP) is a rare autoimmune disorder resulting in progressive conjunctival fibrosis and ocular surface failure leading to sight loss in up to 50%. This study was designed to optimize an ocular surface sampling technique for identification of novel biomarkers associated with disease activity and/or progressive fibrosis.

**Methods:**

Fifty-seven patients with OcMMP underwent detailed examination of conjunctival inflammation and fibrosis using fornix depth measurement. Ocular surface impression cytology (OSIC) to sample superior bulbar conjunctiva combined with flow cytometry (OSIC-flow) profiled infiltrating leukocytes. Profiles were compared with healthy controls (HC) and disease controls (primary Sjögren's syndrome, pSS). Thirty-five OcMMP patients were followed every 3 months for 12 months.

**Results:**

Overall neutrophils were elevated in OcMMP eyes when compared to pSS or HC (109 [18%] neutrophils/impression [NPI]; 2 [0.2%]; 6 [0.8%], respectively [*P* < 0.0001]) and in OcMMP patients with no visible inflammation when compared with HC (44.3 [7.9%]; 5.8 [0.8%]; *P* < 0.05). At 12 months follow-up, 53% of OcMMP eyes progressed, and this was associated with baseline conjunctival neutrophilia (*P* = 0.004). As a potential biomarker, a value of 44 NPI had sensitivity, specificity, and positive predictive values of 75%, 70%, and 73%, respectively. Notably, eyes with no visible inflammation and raised conjunctival neutrophils were more likely to progress and have a greater degree of conjunctival shrinkage compared to those without raised neutrophils.

**Conclusions:**

These data suggest that OSIC-flow cytometric analyses may facilitate repeated patient sampling. Neutrophils may act as a biomarker for monitoring disease activity, progressive fibrosis, and response to therapy in OcMMP even when the eye appears clinically uninflamed.

Ocular mucous membrane pemphigoid (OcMMP) is an autoimmune disease characterized by recurrent blistering of mucosal surfaces including the skin, typified in the eye by conjunctival inflammation and aberrant tissue regeneration involving excessive scar formation and sight loss in up to 50% from limbal epithelial stem cell failure, corneal scarring, and neovascularization.^1^ Ocular MMP is a rare disorder with a minimum incidence estimated at approximately 0.8 to 1.6/million/year^2^ and a mean age of onset of 65 years, but it may occur with a more aggressive phenotype in younger patients,^3^ with up to 30% not responding to immunomodulatory therapy^4^ and 40% demonstrating progressive conjunctival fibrosis in the absence of clinically detectable ocular surface inflammation leading to delayed diagnosis.^5^

In acute disease, the inflammatory process in OcMMP is manifest as conjunctival inflammation characterized by redness, edema, limbitis, and pain to varying degrees.^4,6,7^ In this situation, clinicians rely upon subjective clinical quantification of disease activity (redness, edema, and so on), usually arbitrarily (as no formal scales exist), into none, mild, moderate, or severe to monitor inflammation and to guide immunosuppressive therapy.^3,4^ Unfortunately, conjunctival scarring frequently progresses even when subjectively, the eye is visibly uninflamed.^4,5^ This suggests either that there is a self-perpetuating fibrotic process in the absence of inflammation or, alternatively, that at a molecular level, persistent inflammation results in progressive conjunctival fibrosis. At present, no biological test or marker exists to quantify the extent of inflammation or detect significantly active inflammatory tissue cascades that represent a prelude to conjunctival fibrosis and ocular surface failure and that may facilitate development and implementation of effective therapeutic protocols designed to successfully and effectively induce, consolidate, and maintain disease remission.

The underlying disease process in OcMMP is driven by autoantibodies against the hemidesmosome subunits (including epiligrin [subunit of laminin 5], bullous pemphigoid antigen 1 [BP230], and bullous pemphigoid antigen 2 [BP180]) at the basement membrane zone (BMZ).^1^ This in turn leads to complement activation and the accumulation of inflammatory cells in the stroma.^8,9^ The CD4^+^ infiltrate is thought to be Th2 in phenotype, associated with activation of TGF-β and IL-13.^10,11^ The differences in the cellular infiltrate between clinically involved and seemingly uninvolved conjunctival mucous membranes are, however, not fully understood.^8,9,12^ While numerous in vitro and ex vivo studies have been undertaken, a major limitation of these is that they represent a “snapshot” of what is happening rather than providing data on disease course that may guide therapeutics. As longitudinal tissue sampling by repeated ocular mucosal tissue biopsy is associated with the risk of precipitating or aggravating disease,^1^ published studies to date are dependent on utilizing tissue “surplus to clinical requirement,” that is, use of residual tissue from diagnostic conjunctival biopsy material, for investigative immunohistochemical cellular phenotyping and quantification.

These technical issues may be overcome by the use of conjunctival ocular surface impression cytology (OSIC) whereby the suprabasement membrane epithelium and cells are removed with a polyethersulfone filter. Ocular surface impression cytology is a simple minimally invasive test currently used in clinical practice for the diagnosis of limbal epithelial stem cell failure.^13^ When adhered cells are recovered and analyzed by multicolor flow cytometry (OSIC-flow),^14–16^ the technique provides a means to characterize the ocular mucosal cellular response associated with progressive conjunctival fibrosis and identify cellular subsets that may be associated with disease activity and progressive fibrosis when clinical evidence of inflammation has subsided.

In this study, we have used OSIC-flow cytometric analyses to phenotype patients with OcMMP and compared them with disease controls (primary Sjögren's syndrome, a condition associated with slowly progressive conjunctival scarring) and healthy age-matched controls. We have shown that the conjunctival epithelial cellular infiltrate in OcMMP is characterized by a significant increase in neutrophils at baseline and decrease in the dominant CD8αβ population. A direct correlation between the neutrophil infiltrate and visible clinical inflammation together with a persistent conjunctival neutrophilia in clinically uninflamed eyes is seen, and this perpetuates fibrotic signaling cascades as exemplified by clinically measurable progression of fibrosis. Ocular surface impression cytology is a simple repeatable technique that allows monitoring of disease course. The presence of conjunctival epithelial neutrophils may represent a disease “biomarker” in patients with OcMMP for the prediction of disease progression and for gauging and optimizing effective therapeutic protocols to prevent blinding scarring and ocular surface failure.

## Methods

### Study Subjects

Clinical data collection and patient sampling were undertaken following ethical approval in accordance with the Declaration of Helsinki (Birmingham East, North and Solihull Ethics Committee: Inflammation in Ocular Surface Disease IOSD 08/H1206/165, UKCRN 7448).

Fifty-seven patients with OcMMP were recruited from patients presenting to the Ocular Surface Disease clinic at the Birmingham and Midland Eye Centre (BMEC) over a 24-month period. The median age was 72 (range, 49–97 years); patients included 30 females (53%); 55 white, and 1 black as defined by the U.S. Census Bureau criteria (http://www.census.gov/topics/population/race/about.html [in the public domain]), and 1 South Asian (Asian or Asian British as defined by the UK Census 2011).^17^ Diagnosis of OcMMP was based on clinical findings characteristic for the disease, namely, progressive conjunctival cicatrization in the absence of other causes of conjunctival scarring.^18–22^ If patients did not have a previous positive tissue biopsy, a confirmatory perilesional conjunctival and/or oral mucosal biopsy for direct immunofluorescence was undertaken. A positive result was defined as linear deposition of IgG, IgA, or complement (C3) along the BMZ.^1^ If typical clinical characteristics were evident, a negative result did not exclude the diagnosis^4,18–20^ because of the recognition of a subgroup of ocular patients who have ocular features consistent with OcMMP but have a negative biopsy.^2,19,21^ In accordance with the first international consensus, a positive indirect immunofluorescence was not an essential requirement for diagnosis.^1^

Cross-sectional comparisons were made with (1) a group of healthy, age-matched controls (HC; *n* = 21; median age 64 [range, 45–83 years]; 12 females; 14 white, 7 South Asian), defined as subjects with no history or clinical evidence of ocular, systemic inflammatory, or autoimmune disease (including dry eye),^22,23^ contact lens wear, previous ocular surgery, cataract surgery within 3 months, or use of topical ophthalmic medication; and (2) a disease control group, primary Sjögren's syndrome (pSS), an autoimmune disease characterized by dry eye disease that is associated with a slowly progressive cicatrizing conjunctivitis. Diagnosis of pSS was based on the revised American–European Consensus Group (AECG) criteria,^24^ and the group comprised 19 patients (18 female; median age 64 [range, 56–79]; 18 white, 1 South Asian). There was no significant difference in age among the three groups.

Thirty-five of the OcMMP patients from the cross-sectional OcMMP cohort were enrolled for longitudinal follow-up with data collected at 0-, 3-, 6-, 9-, and 12-month visits.

### Disease Grading and Staging

LogMAR visual acuity (VA) was recorded and classified as good (0.0–0.5) or in accordance with the WHO definitions of visual impairment (0.5–1.0), severe visual impairment (1.0–1.3), and blind (<1.3).

Disease activity was based upon the extent of conjunctival inflammation after secondary causes of inflammation (infection, trichiasis, and so on) had been excluded: absent, mild, moderate, or severe (severe defined as being inflamed in all four quadrants, the presence of limbitis and/or conjunctival ulceration).^25^ The absence of inflammation was defined as no clinically identifiable conjunctival inflammation on slit-lamp examination.^4^ The stage of disease was determined objectively by using a validated fornix depth measurer (FDM) for the lower and upper fornix.^26^ For comparison, the staging systems described by Mondino and Brown^27^ ([I] 0%–25%, [II] 25%–50%, [III] 50%–75%; 75%–100% loss of inferior fornix) and Foster^28^ ([I] subconjunctival scarring and fibrosis, [II] fornix foreshortening of any degree, [III] presence of any degree of symblepharon end-stage cicatricial pemphigoid), and Tauber et al.'s^29^ proposed staging system combined with the use of the FDM for determining percentage shrinkage of the lower fornix were used. Progression was defined as an increase in forniceal shrinkage (1 mm or greater) or by Tauber staging. Immunosuppression strategies used a “stepladder” approach as previously described.^3^ Information regarding surgical and therapeutic intervention was also recorded.

**Figure 1:**
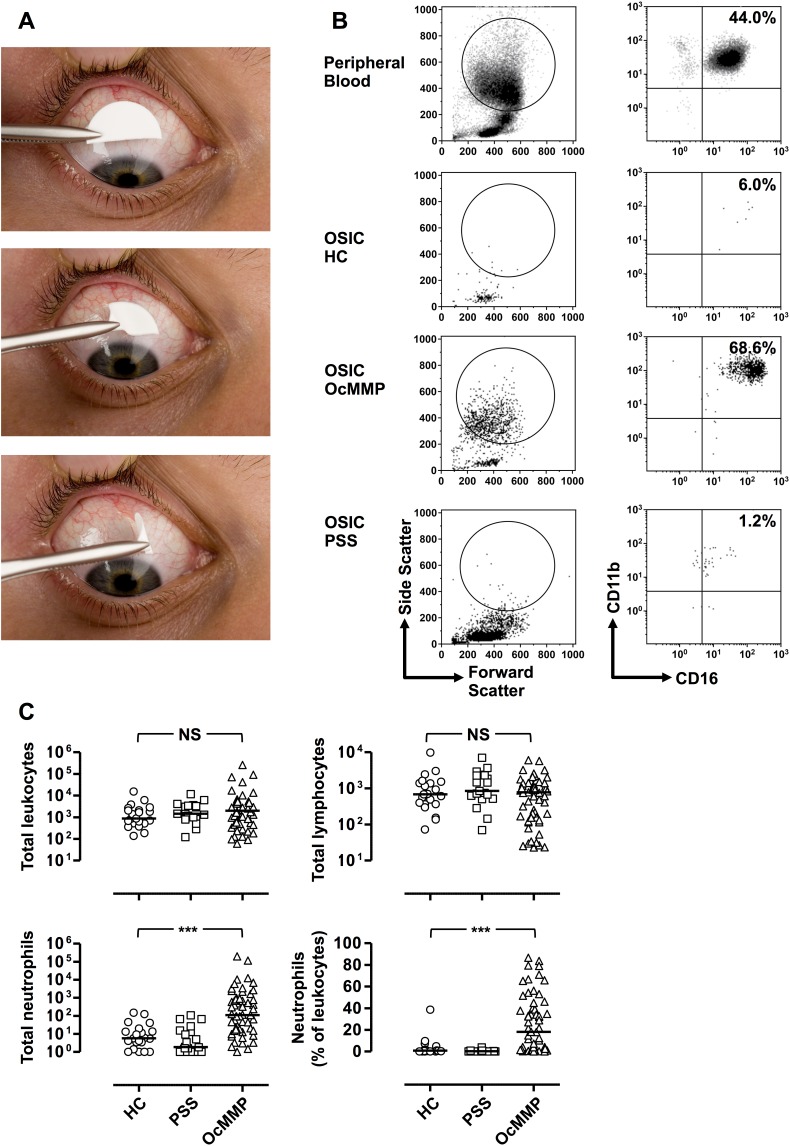
Ocular mucous membrane pemphigoid is characterized by raised conjunctival epithelial neutrophils. (**A**) Ocular surface impression cytology was performed with four semicircle membranes per eye (equivalent to two full impression circles) from an anesthetized superior unexposed bulbar conjunctiva. Semicircles were gently pressed in the conjunctiva for a few seconds and lifted with forceps. (**B**) Representative flow cytometric plots demonstrating the gating strategy to determine conjunctival leukocytes from OSIC and peripheral blood. Live leukocytes were identified by gating for CD45^+^ cells that were negative for the dead cell exclusion dye Sytox blue. Neutrophils were defined as CD45 intermediate, CD14^−^, CD16^+^CD11b^+^ granulocytes. (**C**) OSIC-flow of the OcMMP conjunctival epithelium showed predominant neutrophils compared to healthy controls (HC) and primary Sjögren's syndrome (disease controls, pSS). (Comparisons were undertaken by comparing the most inflamed eye at presentation in patients with OcMMP (*n* = 57) versus the right eye of HC (*n* = 21) and patients with pSS (*n* = 19). Three-group comparisons were undertaken by the Kruskal-Wallis test (**C**) (NS, not significant; *P = 0.01–0.05; **P = 0.001–0.01; ***P < 0.001).

**Figure 2:**
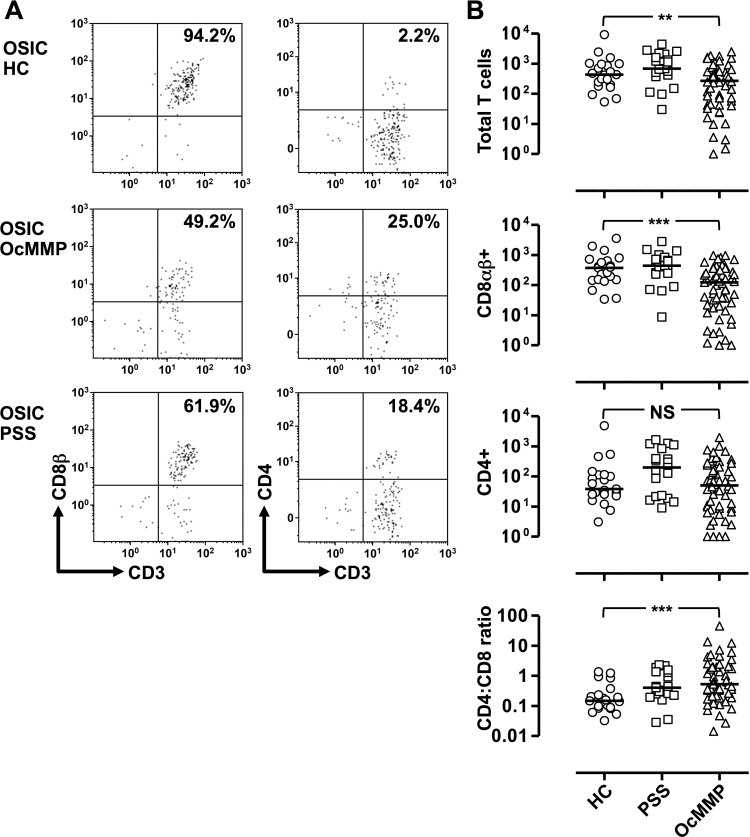
Ocular mucous membrane pemphigoid is characterized by a reduction in the dominant CD8αβ^+^ T-cell population and elevation in the CD4:CD8 ratio. (**A**) Representative plots demonstrating the gating strategy to determine conjunctival leukocytes from ocular surface impression cytology (OSIC). Comparisons were undertaken by comparing the most inflamed eye in patients with ocular mucous membrane pemphigoid (OcMMP, *n* = 57) versus the right eye of healthy controls (HC, *n* = 21) and patients with primary Sjögren's syndrome (pSS, *n* = 19) (**B**). Three-group comparisons were undertaken by the Kruskal-Wallis test (NS, not significant; *P = 0.01–0.05; **P = 0.001–0.01; ***P < 0.001).

**Figure 3:**
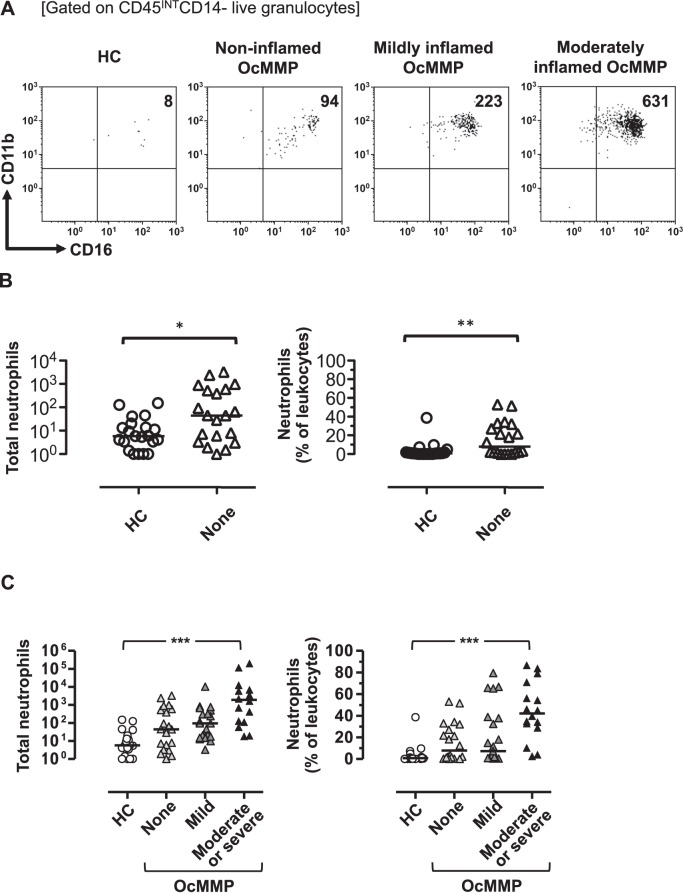
Epithelial neutrophils are present even in clinically uninflamed eyes despite the severity of clinically identifiable conjunctival inflammation correlating with the inflammatory infiltrate. (**A**) Representative flow plots for CD45^INT^CD14^−^ live granulocytes for a healthy individual, a patient with a clinically uninflamed eye, one with mild, and one with moderate clinical inflammation. Healthy and clinically uninflamed individuals were also compared ([**B**] OcMMP *n* = 20, HC *n* = 21). The number and percentages of neutrophils were correlated with the degree of conjunctival inflammation and healthy participants (**C**). Two-group comparison with the Mann-Whitney *U* test and ordinal group comparisons were undertaken by the Jonckheere-Terpstra test (NS, not significant; *P = 0.01–0.05; **P = 0.001–0.01; ***P < 0.001).

**Figure 4:**
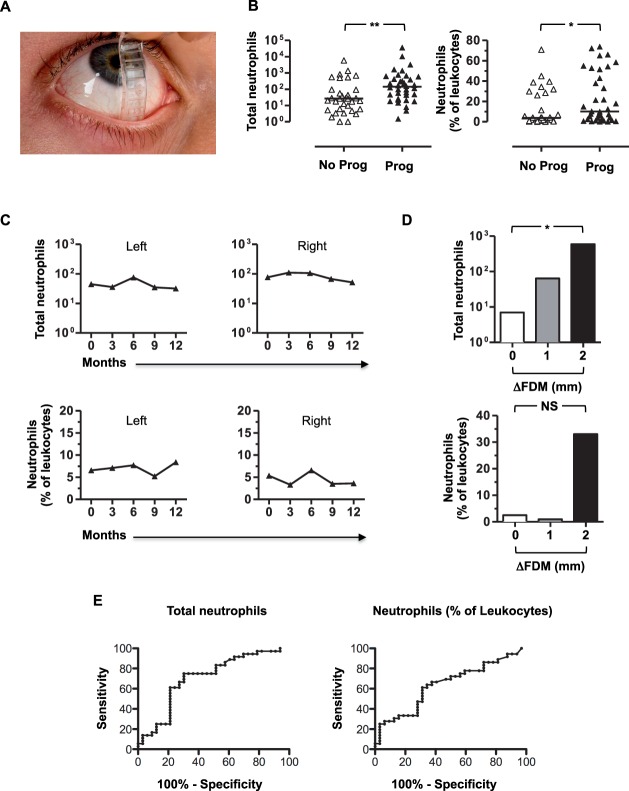
Neutrophil numbers and percentage are significantly higher among eyes that develop lower fornix shrinkage in OcMMP, are maintained over time; and in clinically uninflamed eyes, the number of neutrophils correlates with the degree of central lower fornix shrinkage in OcMMP. Assessment of central lower fornix with a fornix depth measurer (FDM) is shown in (**A**). Comparisons between the conjunctival CD45^INT^CD11b^+^CD16^+^CD14^−^ neutrophil numbers and percentage of leukocytes were undertaken in a cohort of 35 patients with OcMMP by the Mann-Whitney *U* test (NS, not significant; *P = 0.01–0.05; **P = 0.001–0.01). The median neutrophil numbers and percentage are shown for eyes that did not (No Prog) and did progress (Prog) (**B**). Changes over time were calculated by using a generalized estimating equation (GEE) at 0, 3, 6, 9, and 12 months follow-up for right and left eyes. This is represented schematically for the median neutrophil numbers over time ([**C**] *left and right columns*, respectively). The number and percentage of neutrophils were compared by ΔFDM (the change in central lower fornix measured in millimeters between 0 and 12 months; FDM at 12 months − FDM at 0 months) (**D**). ROC curve analysis for neutrophil numbers and percentage are shown in (**E**) (*n* = 69 eyes). Comparisons were undertaken by the Jonckheere-Terpstra test (NS, not significant; *P = 0.01–0.05). FDM, fornix depth measurer.

### Sample Collection and Laboratory Analysis

Both eyes were clinically phenotyped and sampled.

#### Ocular Surface Impression Cytologic Sampling.

Collection of conjunctival cells was undertaken with autoclaved Supor 200 polyethersulfone filters (0.2-μm membranes) (VWR, Lutterworth, UK) divided into two semicircles (measuring 13 × 6.5 mm^2^ each).^30^ Conjunctival OSIC was performed with up to four semicircle membranes per eye (equivalent to two full impression circles) from the superior unexposed bulbar conjunctiva using a sterile technique^30^ (Fig. 1A) and as previously described by our group.^15,16,31^ Conjunctival cells were recovered by gentle agitation and centrifuged (400*g* for 5 minutes), and the supernatant was discarded, leaving 10 μL, which was resuspended in a further 90 μL RPMI:10% Heat Inactivated Fetal Calf Serum (HIFCS) (to a total 100 μL) unless otherwise stated. Cells were placed in 96-well plates for flow-cytometric analysis.

#### Peripheral Blood.

Peripheral blood was collected in EDTA tubes and aliquoted at a volume of 1 mL, centrifuged, and resuspended in 1:10 dilution of filter-sterilized red cell lysis buffer (8.29 g NH_4_Cl [Sigma-Aldrich, Gillingham, Dorset, UK], 1 g KHCO_3_ [Sigma-Aldrich], and 37.2 mg EDTA [Sigma-Aldrich] per liter distilled H_2_O). After 5 minutes at room temperature, the suspension was diluted with up to 15 mL RPMI to block further lysis. Following centrifugation, the pellet was resuspended in phosphate-buffered solution (PBS; Oxoid, Cambridge, UK) at a concentration of 1 × 10^7^ cells/mL and aliquoted at a volume of 100 μL into individual wells.

#### Flow Cytometry.

Flow cytometry was undertaken with a Dako Cyan ADP High Performance flow cytometer (Beckman Coulter, High Wycombe, UK). Multicolor cytometry compensation was performed using cells or compensation beads individually stained with each fluorochrome-conjugated antibody. Compensation circumvents spectral overlap by adjusting for false positives from other fluorochromes. This is achieved by setting compensation levels for one fluorochrome at a level commensurate with the background fluorescence of another, for example, PE-Cy7 anti-CD56 versus PerCP Cy5.5 anti-CD4; the fluorescence intensity for the positive population of CD56-stained beads/cells is adjusted so that it is at the same median fluorescence as background (for the PerCP Cy5.5 channel).

Cells (100 μL) were placed into 96-well plates (with a cell count per well ranging from 2 × 10^5^ to 1 × 10^6^ for peripheral blood mononuclear cells [PBMCs]) or 20 μL positive and negative compensation beads. Cells were centrifuged for 4 minutes at 400*g* at 4°C; the supernatant was removed and the 96-well plate gently vortexed. Cells were stained with surface marker antibodies (made up in 50 μL at appropriate dilutions) and incubated on ice in the dark for 20 minutes. One hundred microliters Fluorescence-activated cell sorting (FACS) buffer (PBS and 0.5% bovine serum albumin [BSA; Sigma-Aldrich] was added to each well prior to further centrifugation and removal of supernatant. Cells were resuspended in 295 μL FACS buffer and 5 μL counting beads (CALTAG/Invitrogen, Paisley, UK) (1002 beads/μL) buffer prior to analysis. For dead cell exclusion, 30 μL Sytox blue (Invitrogen, Paisley, UK) was added at a dilution of 1/800 to the FACS tubes and incubated for 5 minutes prior to running on the flow cytometer.

Cell-surface fluorochrome-labeled monoclonal antibodies were employed in two panels. Panel 1 included mouse anti-human CD45RO (FITC), γδTCR (phycoerythrin, PE), CD4 (PerCP Cy5.5), CD45 (allophycocyanin, APC), CD3 (Alexa Fluor 780) (Ebioscience, Hatfield, UK); CD8α (Pacific Orange) (Invitrogen); CD8β (PE Texas Red) (Beckman Coulter); CD56 (PE Cy7) (Biolegend, Cambridge, UK). Panel 2 included mouse anti-human CD16 (FITC), CD45 (allophycocyanin), CD14 (Alexa Fluor 780) (Ebioscience); CD20 (Pacific Orange), CD19 (PE Texas Red) (Invitrogen); CD138 (PerCP Cy5.5) (BD, Oxford, UK), and CD11b (PE Cy7) (Biolegend). These were titrated to determine the optimal concentrations.

Appropriate panels were applied to cells recovered from conjunctival OSIC or peripheral blood. Gating strategies were as previously described.^15,16,31^

### Statistical Analysis

Data were collected on all eyes and comparisons were undertaken between the worse-affected eye for cross-sectional cohort analysis of inflammation and the better-seeing eye for VA. Cross-sectional comparisons were undertaken by nonparametric Mann-Whitney *U* test for two groups, the Kruskal-Wallis test with Dunn's post hoc analysis when comparing continuous variables, for example, cell numbers or percentage for both eyes. The Jonckheere-Terpstra test was used for ordered groups.

Data for both eyes were used for progression in the longitudinal cohort, as outcomes could be different for each eye for an individual. Initial comparisons were undertaken by nonparametric Mann-Whitney *U* test for two groups. If differences using the Mann-Whitney *U* test were significant, generalized estimating equations (GEEs) were employed when using data for both eyes in order to determine correlation and establish whether progression data for both eyes were significant while taking the effects of correlation into consideration. Normality was assessed using a Kolmogorov-Smirnov test and data were log transformed where necessary. Differences in progression were determined by the Wald χ^2^ test. Differences in repeated measures were also determined by GEE between the right and the left eyes. Overall difference over time was examined by the Huynh-Feldt test in association with repeated measures analysis of variance.

Receiver operating characteristic (ROC) curve analyses were undertaken with sensitivity and specificity for the neutrophil numbers and percentage together with positive predictive values as previously described.^32^ Differences in progression between noninflamed eyes were undertaken by the Jonckheere-Terpstra test or the Fisher's exact test.

Analyses were performed using IBM SPSS 19 (IBM Corp., Armonk, NY, USA) and Prism version 5.0 for Macintosh (GraphPad Software, La Jolla, CA, USA). Statistical significance was defined as *P* < 0.05.

## Results

### Demographic Information

Demographic and baseline clinical details for OcMMP and pSS (disease control group) are summarized in Tables 1 and 2, respectively. The majority of patients with OcMMP were on topical ocular surface therapy (glucocorticoids, preservative-free lubrication) while only 5% (3/57) required topical antiglaucoma medication. Fifty-three percent (30/57) were on some form of systemic immunosuppression at recruitment to the cross-sectional study. Of the 35 patients enrolled into the longitudinal study, 16/35 (46%) were on immunosuppression at baseline, but by 12 months follow-up, their requirement for immunosuppression increased to 71% (25/35). After exclusion of other causes of reduced vision (cataract, glaucoma, uveitis, and optic neuritis; *n* = 10), logMAR VA in the better-seeing eye was measured at 0.0 to <0.5 in 91% (43/47), >0.5 to <1.0 in 4% (2/47), >1.0 to <1.3 in 0%, and >1.3 in 4% (2/47). Patients with OcMMP had levels of dry eye disease comparable to the pSS disease controls defined by tear film break up time (TBUT) (OcMMP median 7 [range, 0–11] seconds versus pSS 5 [2–8] seconds, *P* = 0.09). Primary Sjögren's syndrome patients did not receive glucocorticoids or cyclosporin A at the time of sampling.

**Table 1:**
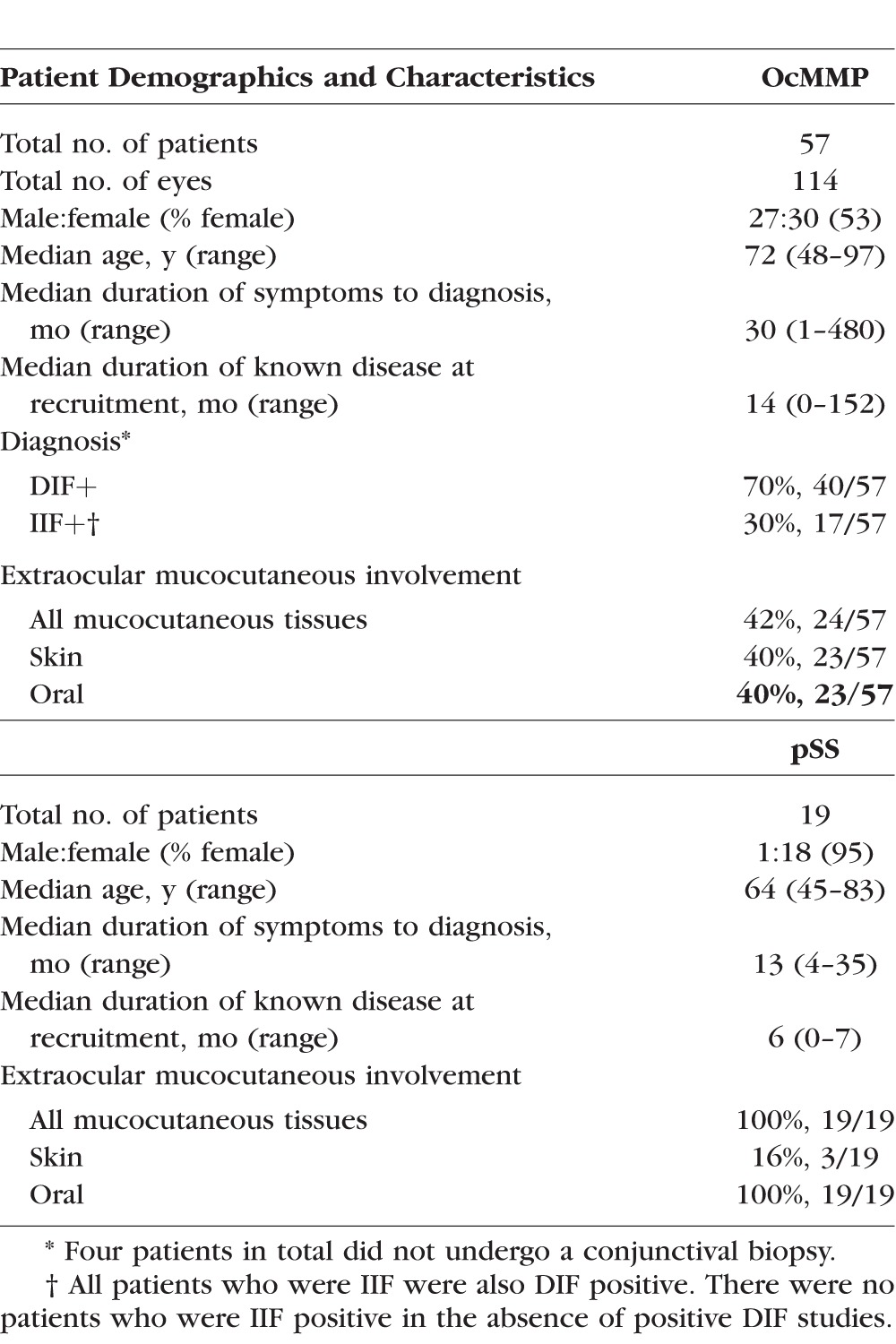
Patient Demographics and Characteristics for the OcMMP and pSS Cohorts at Baseline. Direct Immunofluorescence (DIF) and Indirect Immunofluorescence (IIF) Refer to the Proportion of Patients Who Demonstrated the Linear Deposition of Immunoglobulin G, A, or Complement (C3) Along the Basement Membrane Zone (BMZ) or Had Measurable Titers of Immunoglobulin in the Serum, Respectively

**Table 2:**
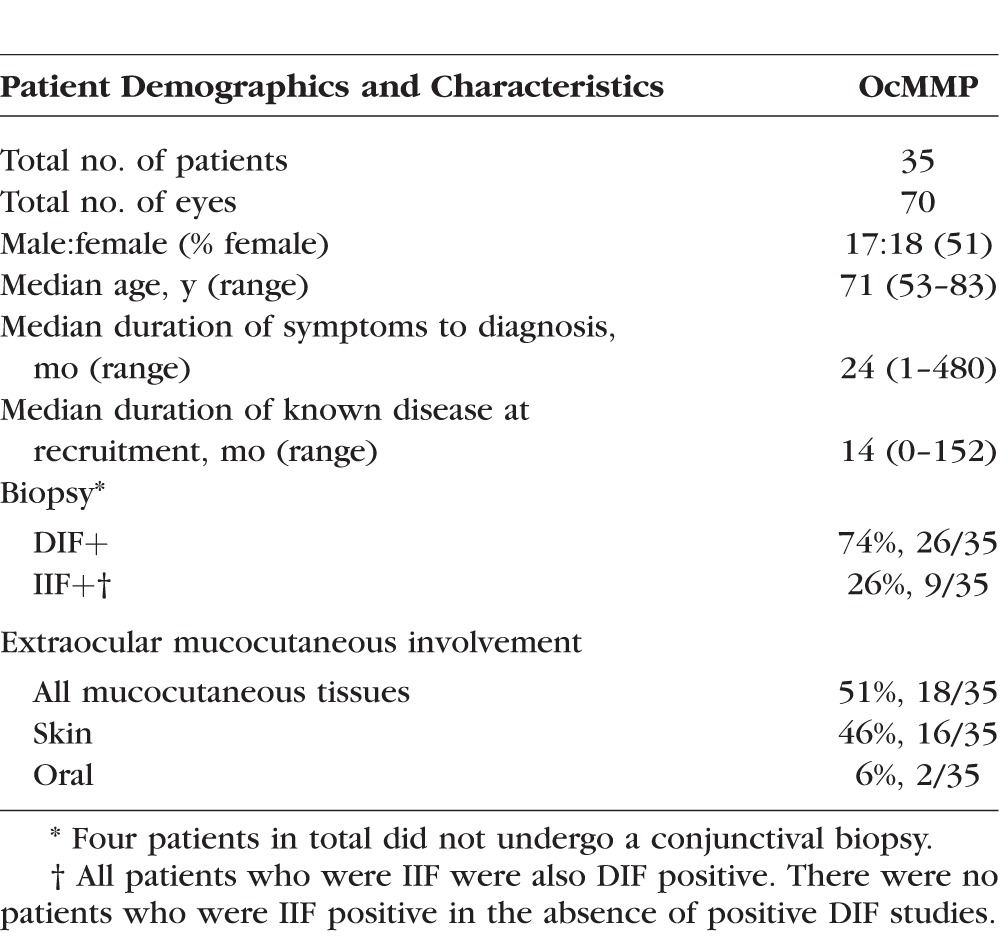
OcMMP Longitudinal Study Cohort Patient Demographics and Characteristics: 35 Patients Were Followed Prospectively and Underwent Clinical Phenotyping Together With OSIC Sampling at 0, 3, 6, 9, and 12 Months. Direct Immunofluorescence (DIF) and Indirect Immunofluorescence (IIF) Refer to the Proportion of Patients Who Demonstrated the Linear Deposition of Immunoglobulin G, A, or Complement (C3) Along the Basement Membrane Zone (BMZ) or Had Measurable Titers of Immunoglobulin in the Serum, Respectively

For the full cohort, at recruitment conjunctival inflammation was present in 65% (37/57) of OcMMP patients, with the remaining (35% [20]) having no visible conjunctival inflammation at baseline. Details of the severity of inflammation together with the damage (scarring) indices derived from clinical data recorded in validated clinical record forms (CRF) are outlined in Supplementary Table S1. Median central lower fornix depth was 4 (range, 0–13) mm; the number of lower fornix symblephara was 2 (0–6), with horizontal symblephara involvement 6 (0–30) mm. Forty-three percent of eyes (49/114) were Mondino stage III/IV and 96% (110/114) were Foster stage III/IV.

### OSIC-Flow of the OcMMP Conjunctival Epithelium Shows Predominant Neutrophils and an Increase in the CD4:CD8 Ratio

Using gating strategies on cells recovered by OSIC-flow as previously described,^15,31^ leukocyte profiles in OcMMP, pSS, and HC were determined in the worst inflamed eye in the OcMMP cohort and right eyes for both healthy (HC) and disease control (pSS) groups. Neutrophils are shown in Figure 1B and lymphocytes in Figure 2A. While overall there was no difference in the total number of leukocytes between groups (Table 3; Fig. 1C), the most striking observation was a significant elevation of conjunctival epithelial neutrophils among patients with OcMMP (neutrophil number/impression [NPI] = 109, 18%) compared to both disease and HC groups (pSS [1.8, 0.2%]; HC [5.8, 0.8%] [*P* < 0.0001]) (Table 3; Fig. 1C). While an expected preponderance of female patients was seen in both the OcMMP and PSS cohort (Table 1), an effect of sex on conjunctival neutrophilia was not observed (OcMMP: males 11.5% [range, 0.2–83.7]; females 19.8% [0.1–86.7]; *P* value 0.97; pSS 18/19 female, statistical analyses not possible).

**Table 3:**
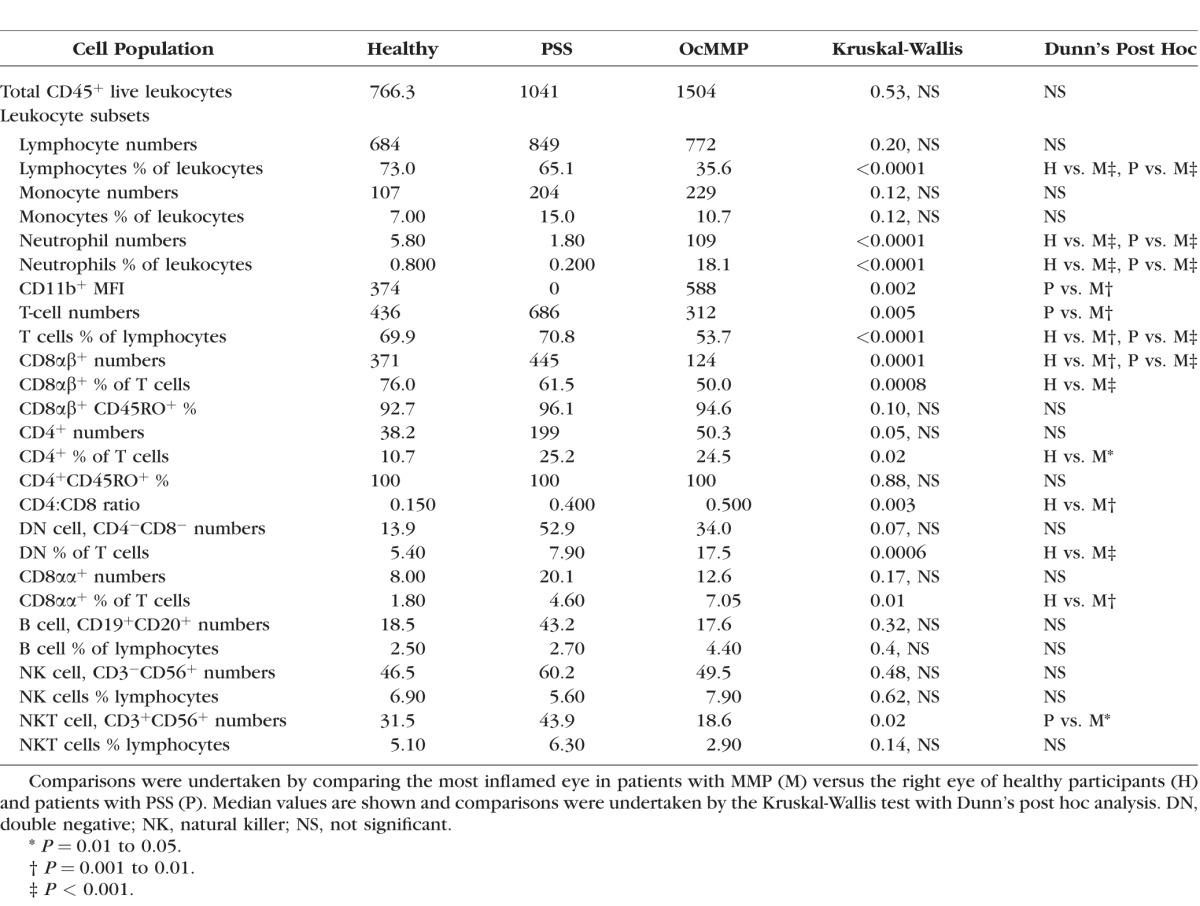
Conjunctival Leukocyte Populations in Healthy Controls, PSS, and OcMMP

Individual lymphocyte subset comparisons demonstrated fewer T cells (CD3^+^CD56^−^ live cells) as a total number and percentage of lymphocytes in the conjunctiva of patients with OcMMP (Table 3). In addition, the dominant CD8αβ^+^ population was significantly lower in patients with OcMMP (HC, 371 versus pSS, 445 versus OcMMP, 124; *P* = 0.0001), resulting in an increased percentage of CD4^+^ cells as a proportion of T cells with a concomitant elevation in the CD4:CD8 ratio in OcMMP compared to HC (0.15 vs. 0.4 vs. 0.5; *P* = 0.003) (Fig. 2B). Despite an expected elevation in CD4^+^ T cells in pSS as previously published,^33^ the CD4:CD8 ratio was greater in OcMMP versus pSS (disease controls), although this difference did not reach statistical significance. An elevation in the percentage of CD8 αβ^+^ and double negative (CD8 αβ^−^CD4^−^) T cells was also observed. No changes in the B cell or NK populations were seen, but a reduction in the Natural Killer T (NKT) cell population was seen in OcMMP compared to HC (Table 3).

There was no difference in the total number/percentage of leukocytes (1579 vs. 2595; *P* = 0.18), monocytes (194 vs. 289; *P* = 0.51), and neutrophils (102 vs. 239; *P* = 0.94) between biopsy-positive (*n* = 40) and -negative (*n* = 17) OcMMP patients. In addition, no differences in lymphocyte and neutrophil populations were observed in the peripheral blood of patients with OcMMP, healthy volunteers, and those with pSS (lymphocytes [37% vs. 44% vs. 35%; *P* = 0.2], neutrophils [52% vs. 45% vs. 49%; *P* = 0.78]), although a difference in the monocyte populations between the pSS and HC group was observed (6% vs. 7% vs. 5%; *P* = 0.04).

### Neutrophils Are Detected in Clinically Uninflamed OcMMP Eyes and Are Associated With the Grade of Visible Conjunctival Inflammation

In clinically noninflamed eyes, the number and percentage of neutrophils were significantly higher when compared to HC 44.3 (7.9%) vs. 5.8 (0.8%); *P* = 0.02 and *P* = 0.004 (Figs. 3A, 3B). This suggests that neutrophils are present even in the absence of clinically manifest, that is, visible conjunctival inflammation. Importantly, a direct association between the neutrophil infiltrate number and percentage is seen across the spectrum of clinical entities defined as a range from HC conjunctiva, OcMMP conjunctiva with no visible inflammation, and OcMMP conjunctiva with visible inflammation graded as mild and moderate/severe (Fig. 3C: HC 5.8 [0.8%] versus noninflamed OcMMP 44.3 [7.9%] versus mildly inflamed OcMMP 94.3 [7.3%] versus moderately/severely inflamed 1912 [42.3%] [*P* < 0.0001]). These data confirm persistence of inflammation occurring at a molecular level in eyes that are clinically classified as uninflamed with no visible inflammation and that neutrophils potentially represent a quantitative measure of conjunctival inflammation whether clinically manifest or not.

### The Effects of Topical Glucocorticoids on Conjunctival Intraepithelial Neutrophils

Systemic glucocorticoids can induce granulocytosis.^34^ As the majority of our cross-sectional patients (54% [31/57] at presentation; 60% ]21/35] at 12 months) were prescribed topical dexamethasone, it was necessary to determine the effect of this synthetic glucocorticoid on the presence of conjunctival neutrophils. This was particularly important given the raised levels of neutrophils in the conjunctival mucosa of patients with OcMMP. An additional cohort of seven healthy volunteers (median age 74 [range, 57–84 years]; four females; six white European, one South Asian) were sampled before and 4 weeks after cataract surgery. Participants were instructed to use topical dexamethasone 0.1% every 6 hours during the postoperative follow-up period. This enabled a comparison of leukocyte numbers in an uninflamed eye with and without the application of topical steroids. The 4-week review samples were taken only if the following criteria were met: Cataract surgery was uncomplicated; there was no evidence of ocular surface or intraocular surface inflammation at 4 weeks post surgery. There was no significant difference in the CD45^INT^CD11b^+^CD16^+^CD14^−^ neutrophil populations before and after the use of topical treatment (13 vs. 0, *P* = 0.06; 1.7% vs. 0%, *P* = 0.13), although the trend suggested a reduction in the numbers present on the ocular surface (Supplementary Fig. S1). We were able to conclude that the use of topical dexamethasone in the OcMMP cohort was unlikely to have contributed to changes in observed in the conjunctival granulocyte population.

### Conjunctival Neutrophils Correlate With Progressive Conjunctival Scarring and Inferior Forniceal Foreshortening Measured With the FDM in OcMMP

A longitudinal cohort of 35 OcMMP patients (Table 2) were followed for a year and reviewed at baseline (Supplementary Table S2) and at 3, 6, 9, and 12 months (Supplementary Table S3). By 12 months, there had been progression of scarring (measured by central depth) in the lower fornix in 53% of eyes, resulting in a median depth of 3 (0–8) mm, representing a significant reduction in lower fornix depth (*P* < 0.001). Similarly, the median central upper fornix depth had contracted in 47% of eyes (*P* < 0.01). The number and extent/width of symblephara had also progressed (number: lower, *P* = 0.02; upper, *P* < 0.001; extent/width: lower, *P* < 0.001; upper, *P* < 0.001); and Tauber scoring had increased in 67% of eyes at 12 months.

Given the raised neutrophils, reduction in CD8 αβ^+^ T cells, and raised CD4:CD8 ratio when comparing MMP to controls (HC and pSS), comparisons among cell populations at baseline and disease progression (measured by an increase in conjunctival scarring) were determined. Neutrophil numbers and percentage were significantly elevated in the group that progressed compared to the group that did not according to lower fornix shrinkage (143 [2–36,636] vs. 27 [1–5910], Mann-Whitney *U* test *P* = 0.004; and 10% [0.1–74] vs. 3.7% [0–71], Mann-Whitney *U* test *P* = 0.04) (Fig. 4B). In light of the significant changes seen in the increased neutrophils (total numbers and percentage), a GEE was undertaken to account for the effects of correlation between the right and left eyes. A moderate degree of correlation was observed between the right and the left eyes (correlation value = 0.376). Furthermore, the number of neutrophils was found to be 128 in the eyes that demonstrated progression of disease versus 39 (*P* < 0.001) in the nonprogression group, confirming the test statistic using the Mann-Whitney *U* test. The percentage of neutrophils was also confirmed as significantly higher among eyes that progressed using the GEE (correlation value = 0.605; 10% vs. 5%; *P* = 0.02). By contrast, the levels of CD8 αβ^+^ T cells and the CD4:CD8 ratio were not different among those with and without evidence of lower fornix shrinkage (139 [39%] vs. 97 [45]; *P* = 0.75 and 0.6 vs. 0.4; *P* = 0.32, respectively).

There was no significant difference in the number/percentage of neutrophils among eyes that progressed versus those that did not progress according to upper fornix depth parameters (49 [4%] vs. 69 [12%]; *P* = 0.56). Similarly, this was also the case for the CD8 αβ^+^ T-cell population (124 [27%] vs. 138 [28%]; *P* = 0.8) and CD4:CD8 ratio (0.5 vs. 0.6; *P* = 0.98), indicating that quantification of neutrophil inflammatory infiltrate, CD8 αβ^+^ T-cell populations, or CD4:CD8 ratios did not predict upper fornix shrinkage during this study period. Comparable findings were seen when assessing progression by Tauber staging in this cohort. Comparing eyes that progressed with those that did not, there was no significant difference in the number/percentage of neutrophils (49 [6%] vs. 41 [4%]; *P* = 0.27), CD8 αβ^+^ T-cell population (114 [40%] vs. 144 [52%]; *P* = 0.36) and CD4:CD8 ratio (0.6 vs. 0.2; *P* = 0.08). In summary, increased neutrophils at baseline were seen in eyes that went on to develop lower fornix shrinkage, as measured with an FDM.

As raised conjunctival epithelial neutrophils seen in patients with OcMMP at baseline were more likely to result in lower lid fornix depth shrinkage, changes over time were examined. In order to verify if this inflammatory infiltrate persisted over time, a GEE was employed. Neutrophil numbers at 0, 3, 6, 9, and 12 months were unaltered, with no significant difference seen over this follow-up period (*P* = 0.46), and no change was observed in neutrophil percentage (*P* = 0.76). A schematic demonstrating the median neutrophil numbers over time for the right and left eyes is shown in Figure 4C. This suggests that the conjunctival inflammatory neutrophil infiltrate is constant in OcMMP and that treatment protocols are suboptimal.

Having determined that an inflammatory infiltrate with neutrophils was present in patients with OcMMP and that this infiltrate was present even among those without evidence of inflammation on clinical examination, it was important to determine whether this finding could predict progression of fibrosis measured by reduction in fornix depth. Subgroup analysis among clinically uninflamed eyes (patients with uninflamed eyes examined at 12 months; *n* = 14) was undertaken, which showed that the change in lower lid fornix depth (ΔFDM) correlated with the number of neutrophils (*P* = 0.035; Jonckheere-Terpstra test) but not by percentage (*P* = 0.85; *P* = 0.52) (Fig. 4D).

In order to determine a cutoff value for inflamed and noninflamed neutrophilia, an increased population of neutrophils in the conjunctiva was defined by using the median number of neutrophils (44) determined in the uninflamed group at cross-sectional analysis at baseline. A count of >44 neutrophils was considered elevated, and those with <44 neutrophils were deemed not to be elevated. As a means of predicting progression among all eyes, the presence of conjunctival neutrophils in OcMMP uninflamed eyes conferred a sensitivity of 75%, a specificity of 70%, and positive predictive value 73%. Neutrophils as a percentage of total leukocytes, that is, 6%, defined a sensitivity of 64%, a specificity of 66%, and positive predictive value of 68%. Receiver operating characteristic curve analysis shows an area of 0.7 (*P* < 0.01) and 0.64 (*P* = 0.046), respectively (Fig. 4E). An alternative means of addressing this question was undertaken by comparing progression among eyes with no inflammation (*n* = 14) with a raised neutrophil count against those without raised neutrophils. Progression rates were found to be significantly higher among those with a greater neutrophil infiltrate (*P* = 0.02; Fisher's exact test), even when uninflamed. These data taken together indicate that progression of conjunctival fibrosis is more likely with an elevation in the number of conjunctival epithelial neutrophils.

## Discussion

“Hidden” conjunctival inflammation in OcMMP provides a major challenge for the clinician. Progressive shrinkage of the conjunctival fornices occurs in up to 50% of patients despite immunosuppression perceived to be adequate in curbing the clinical inflammatory response. In this study, we have demonstrated the presence of a conjunctival epithelial infiltrate characterized predominantly by neutrophils in the ocular mucosa of patients with OcMMP at baseline and shown that persistently raised neutrophils were associated with progressive conjunctival fibrosis, even in the absence of clinically visible inflammation. Importantly, these changes could be detected by longitudinal sampling using a minimally invasive technique, OSIC, currently accepted diagnostically in combination with immunohistochemistry for phenotyping corneal and conjunctival cells in ocular surface metaplasia and failure. Whereas our laboratory outcome measure for determining a biomarker of disease activity and progression was profiling leukocytes with flow cytometry, OSIC opens the arena to other putative laboratory readouts including ocular surface genomics, proteomics, lipidomics, and microbiome analyses. Commercially available devices such as the Eyeprim (Opia Technologies, Paris, France) are now available to simplify OSIC using a Supor 200 polyethersulfone filter and allow nonmedically trained staff to sample the ocular surface.

We analyzed cells recovered from OSIC with flow cytometry to confirm an inflammatory cellular infiltrate within the suprabasement membrane conjunctival epithelium in OcMMP compared to healthy participants. This cellular infiltrate differed from that in patients with pSS who have slowly progressive conjunctival scarring disease. Previous histology-based studies have shown that in conjunctival tissue sections from OcMMP patients who have evidence of minimal but clinically visible inflammation,^8,9,12^ there is predominately a T-cell infiltration of the conjunctival stroma and neutrophils in the epithelium (with a CD4:CD8 ratio of 0.5).^8,9^ These studies also included patients in receipt of topical glucocorticoids at the time of biopsy, suggesting that therapy had not selectively depleted lymphocytes.^8,9^ In severe conjunctival inflammation, the CD4:CD8 ratio increases to 1.0,^8,9^ which reflects an increase in CD4^+^ T cells, a finding that is also seen in human and murine models of pSS.^8,33,35,36^ A murine model does not currently exist for OcMMP, and studies have been restricted to human tissue. A series of 10 conjunctival biopsy samples has confirmed an elevation of Th17 cells in OcMMP compared to HC.^37^ In our study, there was a trend toward raised numbers of CD4^+^ T cells in both MMP and pSS, and the major influence on the increased CD4:CD8 ratio was a reduction in CD8αβ^+^ T cells. We have previously shown that CD8 αβ^+^ T cells are in fact effector memory, mucosal homing, cytotoxic lymphocytes, capable of recognizing herpetic viruses.^16^ Whether this represents an alteration in the effector function of these cells in progressive conjunctival scarring processes such as OcMMP, or simply reflects abnormal architecture of the ocular surface secondary to scarring, remains to be explored. A reduction in CD8αβ^+^ T cells has previously been described in chronic patients with Stevens Johnson Syndrome-Toxic Epidermal Necrolysis (SJS-TEN), although direct comparisons with current and published data are precluded because of the significant age difference between these two disease groups and the fact that conjunctival leukocyte profiles are known to be, in part, dependent on age.^31^

In addition to T cells, increased neutrophils have been reported in tissue analyses of OcMMP patients with severe conjunctival inflammation,^8,9^ and this evidence supports our data examining epithelial infiltrate with OSIC-flow cytometry from clinically inflamed eyes. Importantly, we have also demonstrated an increase in the percentage of neutrophils in OcMMP clinically uninflamed eyes—a finding not seen in either our disease control cohort of patients with pSS (who had equivalent dry eye disease to our OcMMP cohort with minimal conjunctival fibrosis) or HC. It is unlikely that the use of topical steroids or antiglaucoma agents confounded the presence of neutrophils in our cohort. Our validation study on healthy patients administering dexamethasone every 6 hours for 4 weeks demonstrated no increase in conjunctival neutrophil population in the validation time period. It is unlikely that glucocorticoids selectively spared neutrophils by reducing lymphocyte numbers in the OcMMP group. While the percentages were reduced, the increase in neutrophils would account for this, as the absolute numbers of lymphocytes (684 vs. 849 vs. 772; *P* = 0.20 [NS] and leukocytes (766.3 vs. 1041 vs. 1504; *P* = 0.53 [NS]) were consistent among groups. Similarly, it is unlikely that topical antiglaucoma medication had a neutrophil chemoattractant effect, as only three patients in our cohort required ocular hypotensive therapy.

Our data confirm that the neutrophils present in the conjunctival epithelium correlate with the grade of clinical inflammation and persist in an eye without visible conjunctival inflammation. This finding is supported by previous OSIC-flow data in SJS-TEN patients^31^ (who have an OcMMP-like conjunctival scarring phenotype), and tear ELISA analysis, where neutrophil collagenase matrix metalloproteinase (MMP)-8 and -9 together with myeloperoxidase have been detected in patients with both SJS-TEN and OcMMP.^38^ Most importantly, our novel finding demonstrates that eyes with an increase in conjunctival epithelial neutrophils at baseline had evidence of fornix shrinkage at 12 months follow-up. The presence of neutrophils could potentially perpetuate fibrotic signaling cascades and support a role for innate inflammatory cells as a basis for progression of disease in conjunctival scarring disorders^39^ or shaping the adaptive immune response.^40^

The importance of the complement-mediated component in the inflammatory process in OcMMP has previously been emphasized,^41^ with antibodies inducing the classical complement cascade via C3, leading to chronic recruitment of neutrophils to the conjunctival BMZ. The precise reason for neutrophil accumulation in the conjunctiva of patients with OcMMP as opposed to pSS is unclear. In both conditions, dysregulation of CD4^+^ T cells, including an elevation of IL-17 subsets, is seen.^37,42^ In OcMMP, the complement cascade is activated at the BMZ in response to autoantibodies. Presumably this involves the classical pathway via IgG binding to C1q, resulting in the BMZ being coated in C3b. Although murine models suggested that the complement cascade was not involved in subepithelial lesions in skin, closer scrutiny of the mouse conjunctiva has not been undertaken.^43^ The murine ocular surface may or may not have had evidence of blistering, but in humans, this again is not a prominent feature; scarring takes place without gross evidence of blister formation. It is therefore quite possible that autoimmune-mediated recruitment of complement contributes to the inflammatory infiltrate in turn by recruiting neutrophils. This may be compounded by recruitment of other cells to the inflammatory environment within the conjunctiva as in other mucosal sites. Indeed, CD4^+^ T cells have been shown to regulate neutrophil recruitment to the site of inflammation in a mouse colitis model.^44^

The absence of detectable coexisting infection suggests that this may be a potential mechanism for chronic neutrophil elevation or an undetectable alteration in the diseased ocular microbiome driving the immune response.^45^ In a fungal model of keratitis, neutrophils demonstrate increased elastase and MMP-9 activity via autocrine production of IL-17 in response to IL-6 and IL-23.^46,47^ In addition, in cystic fibrosis, pseudomonal infection induces a persistent elevated inflammatory response to IL-17. Abrogating IL-17 prior to infection in mice has been shown to improve outcome, suggesting hyperresponsiveness to infection in the airways of patients and consequent damage via neutrophils.^48^ Bullous pemphigoid (an immunobullous disease affecting the skin), shares target antigens to circulating autoantibodies in the BMZ, for example, BP180.^1,49,50^ Neutrophil elastase induces subepithelial blisters in a murine model of this disease, with elastase-deficient mice being resistant to subepithelial blistering.^51^ Neutrophil serine proteases may also play a role in fibrosis. Mice lacking neutrophil elastase, for example, have been shown to be resistant to pulmonary fibrosis^52^; and in cryptogenic fibrosing alveolitis, an elevation of neutrophil numbers and elastase is seen in tissue compared to the clinically less severe fibrotic process evident in systemic sclerosis.^53^ The neutrophil collagenase MMP-8 has also been shown to induce fibrosis in a bleomycin lung injury model,^54^ and neutrophil extracellular trap (NET) formation in respiratory mucosa has been associated with lung myofibroblast differentiation and scar formation.^55^ Neutropenic mice have been shown to have low scarring potential and accelerated wound healing.^56^ Furthermore, the presence of NETs has been detected in ocular surface disease.^57^ Together, these data suggest the putative role for neutrophils in profibrotic inflammatory processes that have the potential to persist and drive adaptive immune response in OcMMP. The relative contributions of an altered ocular surface microbiome secondary to cicatrization, autoimmune-driven complement activation of neutrophils, or both have yet to be determined.

Limitations in this study include an inability to account for different immunosuppressive strategies on the effects of neutrophils (these included prednisolone, dapsone, mycophenolate, methotrexate, rituximab, cyclophosphamide, and combinations thereof) over the study period. Subgroup comparison and correlation with progression and neutrophil numbers were therefore beyond the scope of this study. It would also be interesting in future validation studies to attempt quantification and potentially include a positive control group that facilitates studying the effect of topical glucocorticoids before and after their introduction in conjunctival inflammation.

These data, however, confirm that subclinical inflammation takes place in OcMMP, sometimes despite the introduction of systemic immunosuppression, irrespective of its mode of action. This inflammatory process is characterized by a conjunctival neutrophilia, and this can be determined in a minimally invasive fashion through OSIC-flow. An increase in conjunctival neutrophils confers a greater chance of disease progression despite a failure to determine disease activity at a clinical level, that is, through observing conjunctival hyperemia. This failure to detect this inflammatory process facilitates a chronic, even low-grade, elevation in neutrophils. Crucially it is the inability to circumvent neutrophil elevation that promotes fibrosis. The maintenance of an otherwise undetectable underlying inflammatory process can lead to cicatrization, irrespective of a further elevation during the course of the study.

In summary, our data show the potential for OSIC combined with multicolor flow cytometry to determine epithelial neutrophils as a disease biomarker,^58^ with a positive predictive value in the region of 70%. Having a minimally invasive test available in the clinic to detect “invisible” ocular surface inflammation could provide a major improvement in monitoring disease activity, especially when considering patients who have a clinically uninflamed eye. Selected and effective immunomodulatory targeting of patients at greater risk of progressive conjunctival scarring based upon detection and quantification of neutrophils could not only provide cost-effective biomarker-guided treatment but also hugely benefit patients who have potentially blinding OcMMP. Neutrophil-specific or persistent innate response drug targeting may be advantageous in the future development of therapeutics for the management of OcMMP.

## Supplementary Material

Supplement 1

Supplement 2

Supplement 3
