# Supplementary material for: Conjunctival Neutrophils Predict Progressive Scarring in Ocular Mucous Membrane Pemphigoid
Source: Invest Ophthalmol Vis Sci. 2016 Oct;57(13):5457–69. doi: 10.1167/iovs.16-19247 (PMC5072540; doi:10.1167/iovs.16-19247)
Supplement: Supplement 1 [file iovs-57-11-18_s01.docx]

**Supplementary Tables**

| **Disease Activity** | **Right Eyes** | **Left Eyes** | **All Eyes** |
| --- | --- | --- | --- |
| **Conjunctival Inflammation** |  |  |  |
| Absent | 40% (23/57) | 42% (24/57) | 41% (47/114) |
| Mild | 39% (22/57) | 35% (20/57) | 37% (42/114) |
| Moderate | 16% (9/57) | 19% (11/57) | 18% (20/114) |
| Severe | 5% (3/57) | 3.5% (2/57) | 4% (5/114) |
|  |  |  |  |
| **Disease Damage** | **Right eyes** | **Left eyes** | **All eyes** |
| **Upper Fornix** |  |  |  |
| Depth in mm (median[range]) | 14mm[0-22] | 13mm[0-22] | 14mm[0-22] |
| Symblephara number (median[range]) | 0[0-3] | 0[0-4] | 0[0-4] |
| Symblephara involvement (Horizontal;median[range]) | 0[0-15] | 0[0-15] | 0[0-15] |
|  |  |  |  |
| **Lower Fornix** |  |  |  |
| Depth in mm (median[range]) | 4mm[0-13] | 4mm[0-12] | 4mm[0-13] |
| Symblephara number (median[range]) | 2[0-6] | 2[0-5] | 2[0-6] |
| Symblephara involvement (Horizontal;median[range]) | 8[0-30] | 6[0-30] | 6[0-30] |
| Mondino |  |  |  |
| I | 12% (7/57) | 12% (7/57) | 12% (14/114) |
| II | 26% (15/57) | 26% (15/57) | 26% (30/114) |
| III | 35% (20/57) | 39% (22/57) | 37% (42/114) |
| IV | 26% (15/57) | 23% (13/57) | 25% (28/114) |
| Foster |  |  |  |
| I | 1.8% (1/57) | 1.8% (1/57) | 1.8% (2/114) |
| II | 10.5% (6/57) | 10.5% (6/57) | 10.5% (12/114) |
| III | 65% (37/57) | 61% (35/57) | 63% (72/114) |
| IV | 23% (13/57) | 26% (15/57) | 25% (28/114) |
| Tauber |  |  |  |
| I | 1.8% (1/57) | 1.8% (1/57) | 1.8% (2/114) |
| IIa | 4% (2/57) | 5% (3/57) | 4% (5/114) |
| IIb | 1.8% (1/57) | 1.8% (1/57) | 1.8% (2/114) |
| IIc | 5.3% (3/57) | 4% (2/57) | 4% (5/114) |
| IId | 0% (0/57) | 0% (0/57) | 0% (0/114) |
| IIIa | 26% (15/57) | 26% (15/57) | 26% (30/114) |
| IIIb | 28% (16/57) | 25% (14/57) | 26% (30/114) |
| IIIc | 7% (4/57) | 7% (4/57) | 7% (8/114) |
| IIId | 1.8% (1/57) | 0% (0/57) | 1% (1/114) |
| IV | 23% (13/57) | 28% (16/57) | 25% (29/114) |

Supplementary Table 1: Disease Activity (Clinically visible conjunctival inflammation) and Disease Damage: Primary measures of scarring at baseline in the OcMMP study.. Conjunctival inflammation was graded by the scale described by Elder *et al* (26). Stratification of patients by upper and lower fornix scarring. Symblephara involvement is the percentage of the horizontal fornix obliterated by symblephara as measured by fornix depth measurer (FDM). Primary measures were considered to be upper fornix depth, lower fornix depth and lower fornix Tauber scoring and summarized by the highest point in the scale (30). Mondino and Foster scales are shown for comparative purposes (28,29).

| **Disease Activity** | **Right Eyes** | **Left Eyes** | | **All Eyes** | |  |
| --- | --- | --- | --- | --- | --- | --- |
| **Conjunctival Inflammation** |  | |  | |  | |
| Absent | 46% (16/35) | | 43% (15/35) | | 44% (31/70) | |
| Mild | 43% (15/35) | | 46% (16/35) | | 44% (47/70) | |
| Moderate | 9% (3/35) | | 9% (3/35) | | 9% (6/70) | |
| Severe | 3% (1/35) | | 3% (1/35) | | 3% (2/70) | |
|  |  | |  | |  | |
| **Disease Damage** | **Right eyes** | | **Left eyes** | | **All eyes** | |
| **Upper Fornix** |  | |  | |  | |
| Depth in mm (median[range]) | 14mm[0-22] | | 13mm[0-22] | | 13.5mm[0-22] | |
| Symblephara number (median[range]) | 0[0-3] | | 0[0-4] | | 0[0-4] | |
| Symblephara involvement (Horizontal;median[range]) | 0[0-15] | | 0[0-15] | | 0[0-15] | |
|  |  | |  | |  | |
| **Lower Fornix** |  | |  | |  | |
| Depth in mm (median[range]) | 4mm[0-13] | | 4mm[0-12] | | 4mm[0-13] | |
| Symblephara number (median[range]) | 2[0-6] | | 2[0-5] | | 2[0-6] | |
| Symblephara involvement (Horizontal;median[range]) | 10[0-28] | | 10[0-24] | | 10[0-28] | |
| Mondino |  | |  | |  | |
| I | 14% (5/35) | | 11% (4/35) | | 12.9% (9/70) | |
| II | 29% (10/35) | | 23% (8/35) | | 25.7% (18/70) | |
| III | 34% (12/35) | | 43% (15/35) | | 38.6% (27/70) | |
| IV | 23% (8/35) | | 23% (8/35) | | 22.9% (16/70) | |
| Foster |  | |  | |  | |
| I | 0% (0/35) | | 0% (0/35) | | 0% (0/70) | |
| II | 5.7% (2/35) | | 2.9% (1/35) | | 4% (3/70) | |
| III | 68.6% (24/35) | | 68.6% (24/35) | | 69% (48/70) | |
| IV | 25.7% (9/35) | | 28.6% (10/35) | | 27% (19/70) | |
| Tauber |  | |  | |  | |
| I | 0% (0/35) | | 0% (0/35) | | 0% (0/70) | |
| IIa | 5.7% (2/35) | | 2.9% (1/35) | | 4% (3/70) | |
| IIb | 0% (0/35) | | 0% (0/35) | | 0% (0/70) | |
| IIc | 0% (0/35) | | 0% (0/35) | | 0% (0/70) | |
| IId | 0% (0/35) | | 0% (0/35) | | 0% (0/70) | |
| IIIa | 28.6% (10/35) | | 34.3% (12/35) | | 31.4% (22/70) | |
| IIIb | 34.3% (12/35) | | 37.1% (13/35) | | 35.7% (25/70) | |
| IIIc | 8.6% (3/35) | | 8.6% (3/35) | | 8.6% (6/70) | |
| IIId | 0% (0/35) | | 0% (0/35) | | 0% (0/70) | |
| IV | 22.9% (8/35) | | 17% (6/35) | | 20% (14/70) | |

Supplementary Table 2: Baseline Disease Activity (Clinically visible conjunctival inflammation) and Disease Damage: Primary measures of scarring at baseline in the OcMMP study for patients followed up at 12 months. Conjunctival inflammation was graded by the scale described by Elder *et al* (21). Stratification of patients by upper and lower fornix scarring. Symblephara involvement is the percentage of the horizontal fornix obliterated by symblephara as measured by fornix depth measurer (FDM). Primary measures were considered to be upper fornix depth, lower fornix depth and lower fornix Tauber scoring and summarized by the highest point in the scale (30).Mondino and Foster scales are shown for comparative purposes (28,29).

| **Disease Damage** |  | **Right eyes** | **Left eyes** | **All eyes** |
| --- | --- | --- | --- | --- |
| **Upper Fornix** |  |  |  |  |
|  | Depth in mm (median[range]) | 14mm[0-19] | 12mm[0-19] | 12mm[0-19] |
|  | Symblephara number (median[range]) | 0[0-3] | 1[0-2] | 1[0-3] |
|  | Symblephara involvement (Horizontal;median[range]) | 0[0-15] | 0[0-30] | 2[0-30] |
|  | Central Depth | 49%(17/35) | 46%(16/35) | 47%(33/70) |
| **Lower Fornix** |  |  |  |  |
|  | Depth in mm (median[range]) | 4mm[0-8] | 3mm[0-8] | 3mm[0-8] |
|  | Symblephara number (median[range]) | 2[0-6] | 3[1-5] | 3[0-6] |
|  | Symblephara involvement (median[range]) | 12[0-30] | 10[3-26] | 12[0-30] |
|  | Central Depth | 51%(18/35) | 54%(19/35) | 53%(37/70) |
|  | Tauber^¶^ | 63%(22/35) | 71%(25/35) | 67%(47/70) |

^¶^  One patient did not have Tauber scoring documented at 12 months

Symblephara involvement is the percentage of the horizontal fornix obliterated by symblephara as measured by fornix depth measurer (FDM).

Supplementary Table 3: Disease progression by primary and secondary outcome measures in the 35 OcMMP patients followed up for 12 months.
