# Supplementary figures and images for: Conjunctival Neutrophils Predict Progressive Scarring in Ocular Mucous Membrane Pemphigoid
Source: Invest Ophthalmol Vis Sci. 2016 Oct;57(13):5457–69. doi: 10.1167/iovs.16-19247 (PMC5072540; doi:10.1167/iovs.16-19247)

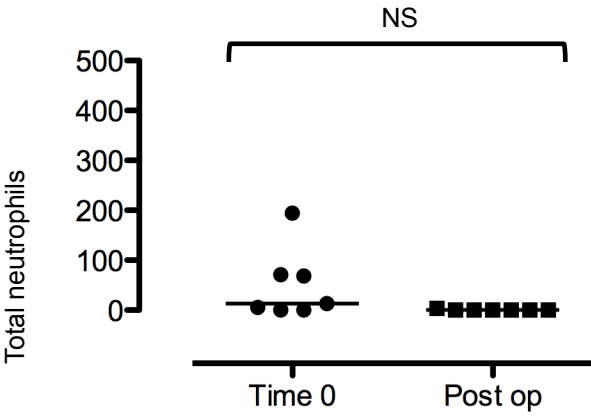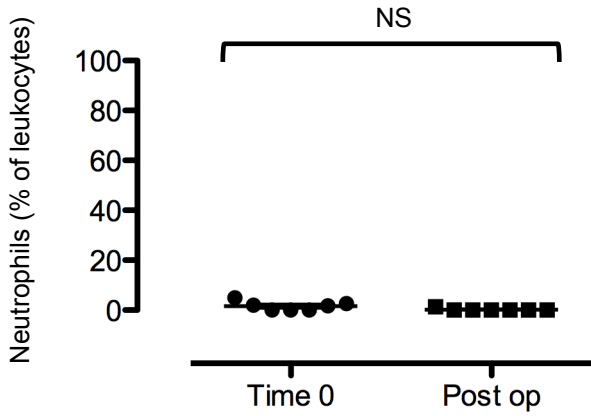

Figure S1

Supplement: Supplement 2 [file iovs-57-11-18_s02.pdf]
