# Supplementary material for: Conjunctival Neutrophils Predict Progressive Scarring in Ocular Mucous Membrane Pemphigoid
Source: Invest Ophthalmol Vis Sci. 2016 Oct;57(13):5457–69. doi: 10.1167/iovs.16-19247 (PMC5072540; doi:10.1167/iovs.16-19247)
Supplement: Supplement 3 [file iovs-57-11-18_s03.pdf]

Supplementary Figure 1. Epithelial neutrophil populations are unaltered by topical Dexamethasone. Neutrophils (defined as $CD45^{INT}$CD11b+CD16+CD14- live granulocytes) are shown as absolute numbers and percentage of leukocytes before and after the use of topical steroids (preservative free Dexamethasone 0.1%). Individuals had conjunctival OSIC before and after uncomplicated cataract surgery. Repeated samples were taken at 4 weeks post-op and in the absence of ocular surface and intra-ocular inflammation (NS: Not significant [p>0.05]).
